# Trajectories of Frequent Short-Term Emergency Department Visits Among Older Adults

**DOI:** 10.7759/cureus.93495

**Published:** 2025-09-29

**Authors:** Rick Mah, Jane McCusker, Eric Belzile, David A Dorr, Deniz Cetin-Sahin, Julia Chabot

**Affiliations:** 1 Department of Emergency Medicine, St. Mary's Hospital Centre, McGill University, Montreal, CAN; 2 Department of Epidemiology, Biostatistics, and Occupational Health, McGill University, Montreal, CAN; 3 St. Mary’s Research Centre, St. Mary's Hospital Centre, McGill University, Montreal, CAN; 4 Department of Medical Informatics &amp; Clinical Epidemiology, School of Medicine, Oregon Health & Science University, Portland, USA; 5 Department of Family Medicine, McGill University, Montreal, CAN; 6 Department of Medicine, St. Mary’s Hospital Centre, McGill University, Montreal, CAN

**Keywords:** ed utilization, emergency department use, frequent ed visits, geriatric emergency care, group-based trajectory modeling, healthcare use patterns, older adults, patient subgroups, resource utilization

## Abstract

Objectives

Frequent emergency department (ED) use is typically defined over a one-year period, but short-term patterns of use among older adults remain poorly understood. We sought to identify distinct trajectories of ED use over a 90-day period and describe their associated patient and visit characteristics, with the goal of informing ED care for this population.

Methods

We conducted a retrospective population study in Quebec, Canada, using provincial administrative databases. Patients aged *≥*65 years with an index ED visit between July 2014 to December 2015 and three or more ED visits in the preceding 90 days were included. Group-based trajectory modeling was used to identify patient groups with distinct trajectories of ED visits; the patient and visit characteristics for each trajectory were compared.

Results

The 10,741 included patients were divided into two cohorts: those with all prior ED visits without admission (No Admission cohort) and those with at least one prior visit resulting in hospital admission (Admission cohort). In both cohorts, two conceptually similar patterns emerged - *Stable *(near-constant probability of ED visits) and *Increasing* (probability rising over time) - although the specific timing and magnitude of changes differed between cohorts. In the No Admission cohort, a third pattern, *Hyperacute* (rapidly rising and high probability of an ED visit near the index visit), was identified and was associated with shorter ED length of stay and fewer chronic conditions compared with other trajectories. The *Increasing* and *Stable* groups showed few differences in patient or visit characteristics apart from certain diagnoses.

Conclusions

This study demonstrates that older adults with frequent ED use can be characterized by distinct 90-day trajectories, which may represent clinically relevant subgroups. Incorporating trajectory-based approaches may enhance understanding of ED utilization patterns and inform strategies to optimize care delivery for this population.

## Introduction

Older adults are important users of the emergency department (ED). In Canada, 17.3% of ED visits in 2003 were by patients 65 years and older, and this has increased to 24.3% in 2021, representing nearly 3.4 million ED visits [1]. These patients are more likely to require admission or die [2] or, if discharged, have a significant risk of ED re-visit, hospital admission, and mortality [3-6]. Older adults are more likely to become frequent users than the general adult population [7]. Studies suggest that up to 6.6% of older patients fall into this category, making up 18% to 38% of ED visits [8-10]. These frequent users are more likely to be admitted, have longer in-patient length of stay, and have increased hospital mortality [11,12].

The most commonly accepted definition of frequent use is four or more visits per year [13]. Few studies have looked at frequent visits over a shorter time interval, even though, for clinicians, this may be concerning as it may represent a missed diagnosis or unmet need. Ronksley et al. studied high-intensity ED use over a seven-day period in the general adult population, but we found no studies on older adults examining high-frequency ED use over such a short time interval [14]. Another identified gap in the literature is how visits by frequent users are distributed over time [13] and whether the pattern of use could define subgroups of frequent users.

The study objectives were to identify trajectories of use among older adults with frequent ED visits over a 90-day period and describe associated patient, ED visit, and hospital characteristics.

These study results were previously presented as a poster at the 18th International Congress of the European Geriatric Medicine Society in September 2022 and the 24th International Conference on Emergency Medicine on May 27, 2025.

## Materials and methods

Design and setting

The research was a retrospective population study using administrative databases from the Quebec Health Insurance Agency and Ministry of Health and Social Services covering all ED visits made by patients insured under the provincial health plan.

Ethics approval for the study was obtained from the Research Ethics Board of the Centre intégré universitaire de santé et de services sociaux de l’Ouest-de-l’Île-de-Montréal, and data access permissions were granted by the Access to Information Commission of Quebec.

Selection of participants and measurements

Subjects included all Quebec patients aged ≥65 years covered under the public health insurance who had an index ED visit between July 2014 and December 2015. The provincial database captures visits to all ED facilities, including those providing only ambulatory care and stabilization prior to transfer; however, visits to these latter facilities were excluded from the analysis. For each patient, an index visit date was assigned or selected randomly using STATA statistical software (uniform distribution) if a patient had more than one visit during the study period. The 90 days before the index visit were used to study and define the time trajectories of ED visits.

Data on each patient’s ED visit were linked to their demographic information using an encrypted identifier. Patient-level variables included age and sex. Visit-level variables included the date of the visit, mode of arrival to the ED (ambulance, ambulatory), triage score (Canadian Triage and Acuity Scale [CTAS] 1-5), presenting complaint (v1.1 Canadian ED Information System), ED placement after triage (stretcher, ambulatory care), discharge diagnosis (ICD-10 10th version), disposition (admit, discharge), and length of stay. Hospital-level variables included ED type (primary, secondary, tertiary) and geographic location (metropolitan, urban/suburban, rural) [15]. The presence of chronic disease (yes/no) for each patient was derived from the Charlson comorbidity index using all ED discharge diagnoses in the three months prior to the index visit [16].

There were little missing data (<0.01%) except for the ICD-10 codes (5.9% of visits did not have a discharge diagnosis).

Data analysis

The outcome used for the trajectory analysis was defined as an ED visit resulting in a hospital admission or an ED visit with discharge. To define a trajectory, only patients with three or more ED visits prior to the index visit were included. To facilitate analyses, patients were separated into those with at least one ED visit leading to hospital admission (Admission cohort) and those without any hospital admissions (No Admission cohort). (Information on the duration of each hospitalization was not available for the Admission cohort.)

Group-based trajectory modeling (GBTM) was used for the No Admission cohort to identify clusters of patients with similar trajectories of ED visits [17-19]. GBTM does not presume discrete sub-populations but rather that the overall distribution of visits can be explained by distinct time trajectories. For the Admission cohort, group-based multi-trajectory modeling (GBMTM), a multivariate extension of GBTM, was applied to examine two-time dependent variables (ED visit with admission and ED visit without admission) simultaneously. The number of trajectories for each cohort was selected using the Bayesian Information Criterion, and model adequacy was evaluated using Nagin’s criteria: average posterior probability of assignment ≥70% and odds of correct classification ≥5 [17,18,20]; full modeling details are provided in the Appendix. A sensitivity analysis was performed to evaluate potential bias from the random selection of the index visit.

For each cohort, the association between the patient and hospital characteristics of ED visits (including the index visit) and trajectories was studied using regression analysis. For the time-dependent variables, all visits were coded and included as covariates. Correlation from multiple ED visits per patient was accounted for by including a patient random intercept within a generalized linear mixed model [21]. For the No Admission cohort, a multinomial distribution was used to handle the three-level trajectory outcome, and a binomial distribution was used for the binary trajectory outcome of the Admission cohort. Odds ratios (ORs) and 95% confidence intervals (CIs) were calculated, with “clinically significant” ORs defined as ≥1.5 or ≤0.67, given the large sample size [22].

For each cohort, we studied the association between discharge diagnosis and the trajectories. We defined one binary variable for each diagnosis (ICD-10) in the preceding 90 days including the index visit (yes/no). Given the large number of diagnoses (>100), we initially applied partial least squares (PLS) regression to the trajectory outcome [23]. Variable importance in projection (VIP), which quantifies the contribution of each variable to the latent PLS components weighted by the extent to which each component accounts for variation in the outcome [23], was used to rank variables. Diagnoses with VIP scores greater than 1.2 were retained for subsequent analysis, followed by nominal logistic regression for the No Admission cohort (three-level trajectories) and binary logistic regression for the Admission cohort [24]. A multivariable model was fitted for each sample, and diagnoses with an OR greater than 1.5 or less than 0.67 were reported.

Analyses were carried out with SAS version 9.4 (SAS Institute Inc., Cary, NC) and STATA version 15.0 (StataCorp, College Station, TX).

## Results

Subject characteristics 

**Figure 1:**
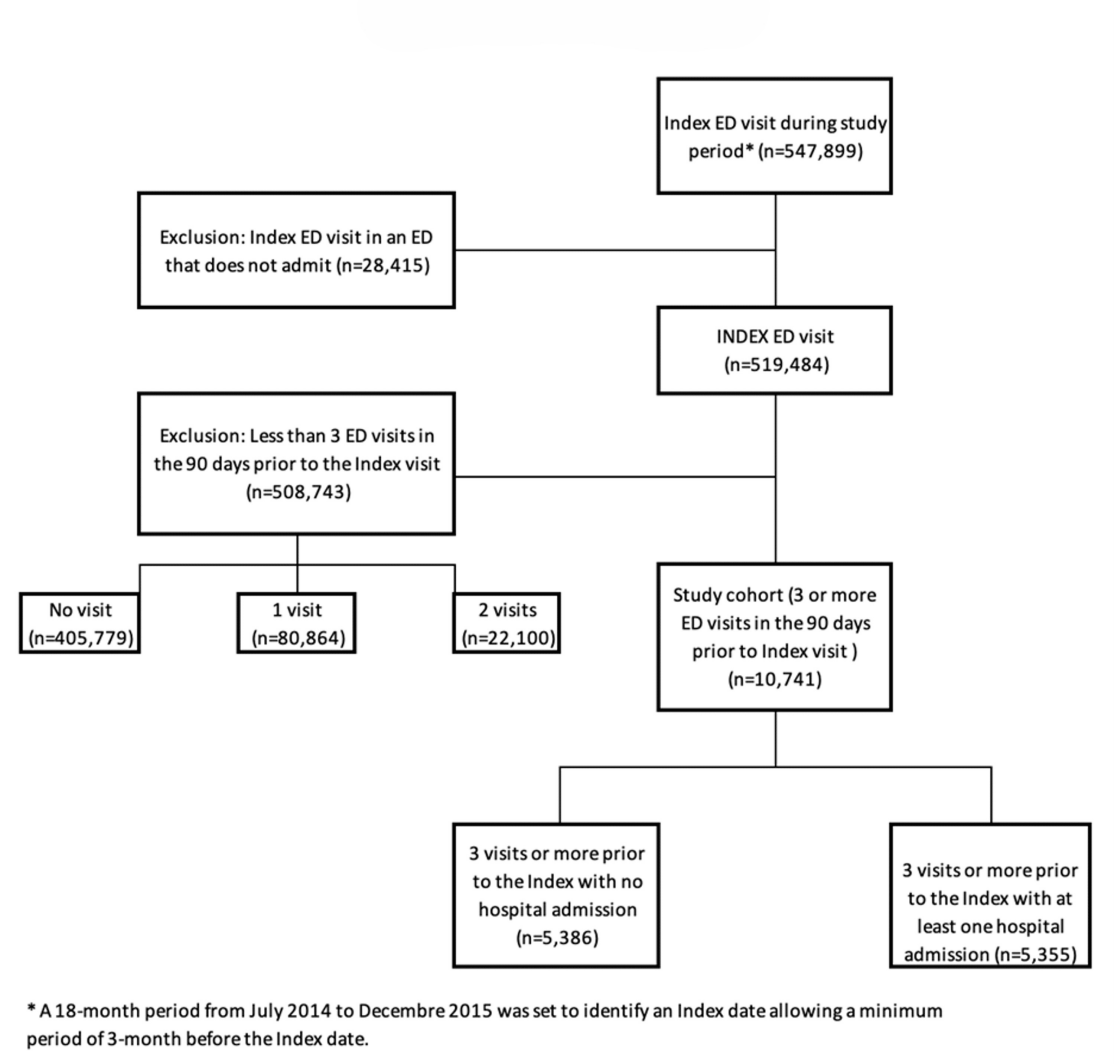
Derivation of study sample ED, emergency department

Of the 519,484 patients aged ≥65 years with an eligible index visit, 2.1% (n=10,741) had three or more visits in the 90 days before the index visit. Including their index visit, these patients accounted for 7.5% (51,029) of all ED visits (684,839) (Figure 1). Table 1 describes the patient characteristics of the Admission and No Admission cohorts. The average number of visits in the 90 days before the index visit was similar for the Admission and No Admission cohorts (3.7 versus 3.8) and for the individual trajectory clusters. For the Admission cohort, there was no difference in the proportion of ED visits with no admission and with admission between the two trajectories.

**Table 1:** Demographic, visit, and hospital characteristics at the index visit; total and by Admission and No Admission sub-samples (n=10,741) Q1, first quartile; Q3, third quartile ¹Système d'information et de gestion des urgences (SIGDU) Normative Framework, Quebec Ministry of Health and Social Services ²Classification according to the Quebec Ministry of Health and Social Services ³Statistical area classification

Variables	Total	No Admission	Admission
	(n=10,741)	(n=5,386)	(n=5,355)
Patient characteristics			
Age, %			
65-74	41%	46%	35%
75-84	38%	37%	39%
85+	21%	16%	26%
Mean (SD)	77.5 (7.8)	76.3 (7.5)	78.7 (8.0)
Female, %	50%	50%	50%
Visit characteristics			
Arrival mode, %			
Ambulance	43%	28%	58%
Walk-in	56%	71%	40%
Other	1%	1%	2%
Triage code, %			
1	2%	1%	3%
2	10%	7%	13%
3	34%	28%	41%
4	33%	35%	31%
5	21%	30%	11%
Chief complaint coded as "return visit"^1^, %	12%	16%	9%
Placement after triage, %			
Stretcher	37%	25%	48%
Ambulatory	63%	75%	52%
Length of stay (hours), median (Q1-Q3)	7.5 (3-21)	5.1 (2-12)	11.6 (5-26)
Chronic disease, n (%)			
No	77%	87%	67%
Yes	23%	13%	33%
Hospital characteristics			
ED classification^2^, %			
Primary	24%	29%	18%
Secondary	45%	42%	49%
Tertiary	31%	29%	33%
Geographic location^3^, %			
Metropolitan	56%	51%	61%
Urban/suburban	26%	27%	25%
Rural	18%	23%	14%

Trajectory model

For the No Admission cohort (n=5,386), results of the GBTM revealed an adequate three- and four-cluster solution. While the four-cluster solution had a better Bayesian information criterion (BIC), for simplicity, the three-cluster solution was retained to conduct the data exploration analyses. For the Admission cohort (n=5,355), the two-cluster solution was retained despite having a slightly lower BIC compared to the three-cluster solution as it was more robust in the sensitivity analysis; see Appendix for analysis and details of model performance. To facilitate the comparison of clusters, the general shape of the trajectory of ED use was described as: *Stable*, *Increasing*, and *Hyperacute*.

**Figure 2:**
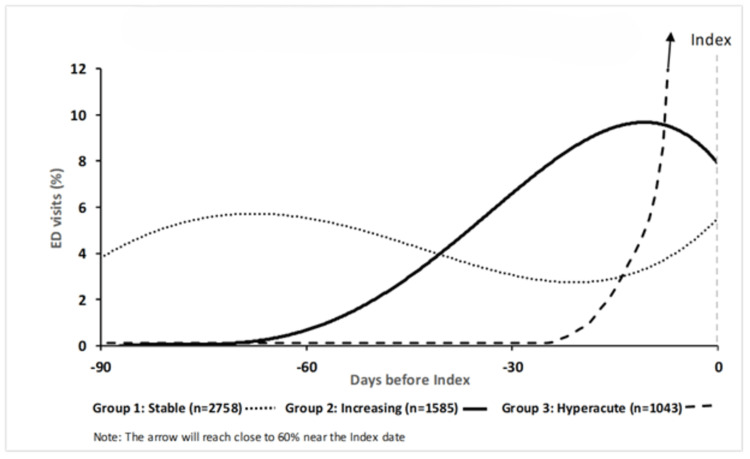
No Admission group trajectories (n=5,386)

The three-trajectory model for the No Admission cohort is presented in Figure 2. The 95% CIs were narrow (not shown). *Stable* (25.7% total sample) demonstrated a constant probability of an ED visit throughout the 90 days, whereas *Increasing* (14.8%) began with a very low probability of a visit that increased approximately 60 days prior to the index visit. *Hyperacute* (9.7%) maintained a very low likelihood of a visit until approximately 20 days prior to the index visit before rapidly increasing to nearly a 60% probability of an ED visit.

**Figure 3:**
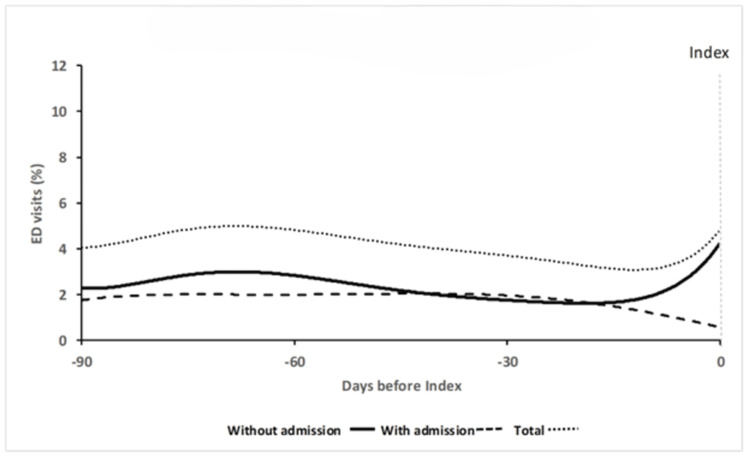
Stable (n=3,618, 67.6%)

**Figure 4:**
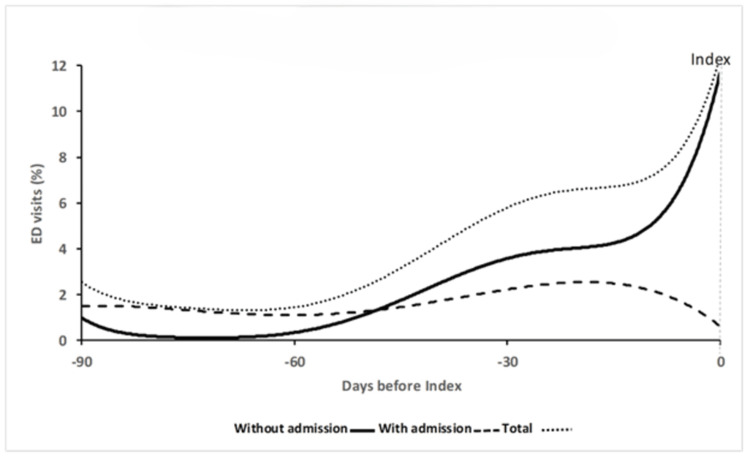
Increasing (n=1,737, 32.4%)

For the Admission cohort, each trajectory cluster was represented by two curves (probability of an ED visit with and without admission) with a cumulative curve to represent the probability of any ED visit (Figures 3, 4). The 95% CIs were narrow (not shown). For the overall probability of an ED visit, *Stable* (33.7% total sample) had near constant probability of a visit, whereas *Increasing* (16.2%) had a lower initial probability which then began to increase at 60 days. When the visit types (i.e., with or without admission) were considered separately, both *Stable *and *Increasing* had a near constant likelihood of an ED visit leading to admission. For ED visits without admission, *Stable* demonstrated a constant probability, whereas the initial likelihood of an ED visit without admission was very low in *Increasing* but then began to increase 60 days prior to the index visit.

Trajectory group patient and visit characteristics 

In the No Admission cohort, there was no difference in the age and sex distribution among the three clusters (Table 2). *Hyperacute* was less likely to have chronic illness and more likely to have a shorter length of stay, more primary level ED use, and visit the same ED compared to *Stable* and *Increasing*. For both the No Admission and Admission cohorts, *Stable *and *Increasing* did not show significant differences in patient, visit, and hospital characteristics.

**Table 2:** Relationship between patient, visit, and hospital characteristics in the 90 days prior to the index visit (including the index visit) and trajectory groups Multivariable model with OR>1.5 or OR<0.67 are in bold *For the time-dependent variables, the patient average proportion was computed Length of stay in hours: No Admission sample (low: <2; medium: 2-8; high: 9+); Admission sample (low: <5; medium: 5-23; high: 24+)

Variables	No Admission (n=5,386)						Admission (n=5,355)		
	Multivariable model: nominal logistic regression						Multivariable model: logistic regression		
	Stable (n=2,758), %	Increasing (n=1,585), %	Hyperacute (n=1,043), %	Increasing vs Stable, OR [95% CI]	Hyperacute vs Stable, OR [95% CI]	Hyperacute vs Increasing, OR [95% CI]	Stable (n=3,618), %	Increasing (n=1,737), %	Increasing vs Stable, OR [95% CI]
Patient/visit characteristics									
Age at index (years)									
65-74	44.1	46.4	52.3	0.98 [0.90; 1.07]	1.09 [0.99; 1.21]	1.11 [0.99; 1.23]	33.1	38.4	1.16 [1.08; 1.24]
75-84	38.5	37.6	34.6	1.02 [0.94; 1.12]	0.94 [0.85; 1.04]	0.92 [0.82; 1.03]	40,0	36.6	0.92 [0.85; 0.99]
85+	17.4	16.0	13.1	1.00	1.00	1.00	26.9	25.00	1.00
Sex									
Female	53.1	48.9	45.7	1,00	1.00	1.00	50.1	50.4	1.00
Male	46.9	51.1	54.3	1.17 [1.11; 1.24]	1.23 [1.15; 1.31]	1.05 [0.98; 1.13]	49.9	49.6	0.98 [0.93; 1.04]
Arrival mode*									
Ambulance	29.0	24.7	12.5	1.00	1.00	1.00	58.4	52.9	1.00
Walk-in	71.0	75.3	87.5	1.22 [1.13; 1.31]	1.79 [1.61; 1.99]	1.47 [1.31; 1.65]	41.6	47.1	1.13 [1.07; 1.20]
Triage code*									
1-3	37.6	34.5	22.1	1.00	1.00	1.00	59.5	56.1	1.00
4-5	62.4	65.5	77.9	1.03 [0.97; 1.11]	1.35 [1.24; 1.47]	1.31 [1.19; 1.43]	40.5	43.9	1.05 [0.99; 1.11]
Chief complaint coded as "return"*									
No	89.8	85.5	79.1	1.00	1.00	1.00	93.2	89.6	1.00
Yes	10.2	14.5	20.9	1.42 [1.31; 1.55]	1.41 [1.28; 1.54]	0.99 [0.90; 1.09]	6.8	10.4	1.40 [1.27; 1.54]
Placement after triage*									
Stretcher	24.5	21.8	11.1	1.00	1.00	1.00	48.6	43.6	1.00
Non-stretcher (ambulatory)	75.5	78.2	88.9	0.98 [0.90; 1.07]	1.24 [1.11; 1.40]	1.27 [1.12; 1.43]	51.4	56.4	1.10 [1.03; 1.16]
Length of stay in ED*									
Low	17.7	20.6	35.2	1.15 [1.05; 1.27]	1.99 [1.79; 2.22]	1.73 [1.54; 1.95]	22.5	26.00	1.16 [1.07; 1.25]
Medium	53.0	53.7	48.7	1.06 [0.99; 1.14]	1.08 [0.98; 1.19]	1.02 [0.92; 1.13]	47.7	48.00	1.08 [1.01; 1.16]
High	29.3	25.7	16.1	1.00	1.00	1.00	29.8	26.00	1.00
Chronic disease									
No	85.0	86.6	94.0	1.00	1.00	1.00	64.8	71.5	1.00
Yes	15.0	13.4	6.0	0.89 [0.82; 0.97]	0.41 [0.36; 0.47]	0.46 [0.40; 0.53]	35.2	28.5	0.76 [0.72; 0.81]
Hospital characteristics									
ED classification*									
Primary	28.2	26.8	40.7	0.93 [0.82; 1.05]	1.59 [1.39; 1.84]	1.72 [1.48; 2.00]	18.1	18.4	1.20 [1.07; 1.35]
Secondary	43.1	43.8	38.5	0.98 [0.91; 1.07]	0.94 [0.85; 1.05]	0.96 [0.86; 1.08]	48.5	49.6	1.09 [1.01; 1.17]
Tertiary	28.7	29.4	20.8	1.00	1.00	1.00	33.4	32.0	1.00
Geographic location*									
Metropolitan	52.2	52.9	39.8	1.00	1.00	1.00	61.8	60.6	1.00
Urban/suburban	26.0	26.6	30.5	0.95 [0.87; 1.03]	1.16 [1.05; 1.28]	1.22 [1.09; 1.36]	23.7	25.6	1.02 [0.95; 1.10]
Rural	21.8	20.5	29.7	0.90 [0.80; 1.02]	0.79 [0.69; 0.90]	0.88 [0.76; 1.01]	14.5	13.8	0.88 [0.79; 0.99]
Hospital change									
No	71.2	74.0	82.7	1.00	1.00	1.00	67.0	63.2	1.00
Yes	28.8	26.0	17.4	0.84 [0.78; 0.90]	0.57 [0.52; 0.62]	0.68 [0.61; 0.74]	33.0	36.8	1.16 [1.09; 1.23]

Trajectory group discharge diagnosis characteristics

In the No Admission cohort, 36% of patients in *Hyperacute* had at least one ED visit for cellulitis, two and three times more frequent than *Stable* and *Increasing*, respectively. Compared to *Hyperacute*, the other two trajectories were more likely to present with atrial fibrillation, chronic obstructive pulmonary disease (COPD) exacerbation, constipation, back pain, urinary tract infection, shortness of breath, and chest pain. Between *Stable *and *Increasing*, *Stable* was more likely to have visits for anemia, fluid overload, COPD, and syncope, whereas* Increasing* was more likely to have follow-up exams as the discharge diagnosis.

In the Admission cohort, visits leading to an admission were studied separately from the no admission visits. Admission diagnoses of COPD, anemia, urinary tract infection, and fever were more likely with *Stable *than with *Increasing*. For visits that did not result in hospital admission, cellulitis was more likely with* Increasing* than *Stable*.

A "missing" category was created to handle the missing discharge diagnoses. When the diagnoses were analyzed in the regression model, the "missing" category did not show any significant association with any outcome.

Table 3 presents the selected diagnoses for the trajectory groups in the No Admission and Admission cohorts.

**Table 3:** Selected diagnoses* for trajectory groups in the No Admission and Admission sub-samples (diagnoses are sorted alphabetically according to the ICD-10) *Diagnoses for this table were selected based on PLS regression, statistical significance, and diagnosis frequency.  Complete analysis is available in the Appendix. **Percentage of patients with at least one visit with the diagnosis. Relevant odds ratios (OR) were defined as OR>1.5 or OR<0.67 from multivariable model regression. Significant OR (at 0.05) are in bold COPD, chronic obstructive pulmonary disease; PLS, partial least squares

	Stable	Increasing	Hyperacute	Increasing than Stable	Hyperacute than Stable	Hyperacute than Increasing
Variables	%**	%**	%**	OR [95%]	OR [95%]	OR [95%]
No Admission (n=5,386)	(n=2,758)	(n=1,585)	(n=1,043)	Nominal logistic regression		
Diagnoses more frequent in Increasing/Hyperacute trajectories				Multivariable model		
Cellulitis (L039)	8.9%	13.8%	36.1%	-	3.81 [3.14; 4.62]	2.67 [2.18; 3.26]
Bursitis, unspecified (M7199)	0.4%	1.1%	1.9%	2.18 [1.01; 4.69]	2.60 [1.19; 5.67]	-
Follow-up exam unspecified treatment (Z099)	2.6%	4.4%	4.5%	1.54 [1.10; 2.16]	-	-
Other specified medical care (Z5188)	8.3%	7.1%	12.6%	-	-	1.62 [1.23; 2.14]
Diagnoses less frequent in Increasing/Hyperacute trajectories						
Anemia (D649)	2.5%	1.4%	0.3%	0.52 [0.32; 0.85]	0.11 [0.03; 0.35]	0.21 [0.06; 0.71]
Fluid overload (E877)	1.7%	0.9%	0.2%	0.52 [0.28; 0.95]	0.13 [0.03; 0.55]	0.25 [0.06; 1.12]
Atrial fibrillation (I480)	2.6%	2.3%	0.7%	-	0.30 [0.13; 0.66]	0.33 [0.15; 0.76]
Bronchitis, acute (J209)	4.2%	3.0%	1.4%	-	0.38 [0.22; 0.67]	0.51 [0.28; 0.92]
COPD with respiratory infection (J440)	3.2%	1.5%	0.6%	0.45 [0.28; 0.72]	0.26 [0.11; 0.61]	0.57 [0.22; 1.45]
COPD exacerbation (J441)	4.4%	4.0%	1.3%	-	0.47 [0.26; 0.85]	0.43 [0.23; 0.80]
Constipation (K590)	4.6%	5.1%	2.2%	-	0.64 [0.40; 1.02]	0.54 [0.34; 0.88]
Back pain (M545)	7.2%	6.3%	3.1%	-	0.47 [0.32; 0.70]	0.55 [0.36; 0.83]
Myalgia (M7919)	1.9%	2.4%	0.4%	-	0.21 [0.07; 0.59]	0.17 [0.06; 0.48]
UTI - Urinary tract infection (N390)	10.5%	9.3%	4.9%	-	0.49 [0.36; 0.67]	0.56 [0.40; 0.78]
Palpitations (R002)	2.3%	1.3%	0.5%	0.63 [0.38; 1.04]	0.29 [0.11; 0.74]	0.46 [0.17; 1.24]
Cough (R05)	3.0%	1.8%	1.0%	0.66 [0.43; 1.02]	0.44 [0.22; 0.87]	-
SOB - Dyspnea (R060)	7.5%	6.9%	1.8%	-	0.37 [0.23; 0.61]	0.36 [0.22; 0.59]
CP - Chest pain (R074)	12.3%	9.3%	4.4%	-	0.43 [0.31; 0.59]	0.56 [0.40; 0.79]
Abdominal pain / Colic (R104)	14.1%	11.2%	6.3%	-	0.54 [0.41; 0.72]	-
Weakness / Fatigue (R53)	8.8%	7.2%	4.2%	-	0.62 [0.44; 0.87]	-
Syncope / Vasovagal (R55)	3.7%	2.3%	1.1%	0.64 [0.43; 0.94]	0.34 [0.18; 0.65]	0.54 [0.27; 1.08]
Open wound lower limb (T131)	0.8%	1.7%	0.7%	-	0.38 [0.16; 0.93]	0.23 [0.10; 0.55]
Admission (n=5,355)	(n=3,618)	(n=1,737)	-	Logistic regression		
Diagnoses for ED visits resulting in an admission				Multivariable model		
Diagnoses more frequent in Increasing trajectory						
Diverticular disease of intestine (K578)	0.5%	1.0%	-	2.03 [1.03; 3.98]	-	-
Diagnoses less frequent in Increasing trajectory						
Clostridium difficile (A047)	2.1%	1.2%	-	0.53 [0.32; 0.86]	-	-
Anemia (D649)	2.7%	1.8%	-	0.64 [0.43; 0.97]	-	-
COPD with respiratory infection (J440)	4.4%	2.5%	-	0.65 [0.46; 0.92]	-	-
COPD exacerbation (J441)	7.1%	4.1%	-	0.59 [0.45; 0.78]	-	-
Urinary tract infection (N390)	3.0%	1.9%	-	0.59 [0.39; 0.87]	-	-
Fever (R509)	2.7%	1.9%	-	0.65 [0.44; 0.97]	-	-
Diagnoses for ED visits without admission				Multivariable model		
Diagnoses more frequent in Increasing trajectory						
Cellulitis (L039)	3.4%	6.0%	-	1.80 [1.38; 2.36]	-	-
Diagnoses less frequent in Increasing trajectory						
Acute upper respiratory infection (J069)	1.5%	0.7%	-	0.46 [0.25; 0.87]	-	-
COPD with respiratory infection (J440)	2.4%	1.4%	-	0.65 [0.41; 1.02]	-	-
Concussion (S060)	1.1%	0.5%	-	0.45 [0.22; 0.94]	-	-

## Discussion

Interpretation

A small group of older adults in the total sample (2.1%) used the ED frequently (7.5% of total visits) over a 90-day period. Nearly half of this group were seen and did not require admission for all of their visits (No Admission cohort). Two trajectories were identified in both the Admission and No Admission cohorts. The *Stable *trajectory demonstrated a near constant probability of an ED visit over the 90-day period, whereas the *Increasing* trajectory began with a very low probability of an ED visit, which then increased towards the index visit. In the No Admission cohort, a third trajectory (*Hyperacute*) was unlikely to visit until 20 days before the index visit and then increased rapidly to nearly a 60% probability of an ED visit. *Hyperacute* had fewer chronic conditions, shorter length of stay, and more primary level ED use. Cellulitis was a frequent and significant diagnosis in this group, whereas the discharge diagnoses observed in *Stable *and *Increasing* were more commonly those for which further investigation (e.g., bloodwork, imaging) or treatment (e.g., analgesia, bronchodilator) would typically be indicated in the ED. This inference reflects the expected clinical management of such conditions rather than observed interventions within this dataset. The patient and visit characteristics of *Increasing* and *Stable *were similar.

Previous studies

To our knowledge, this is the first study to describe frequent ED attenders among older adults based on their temporal pattern of use. Previous research in this population has classified subgroups based on health care utilization and/or patient characteristics [25-27]. These studies identified a subgroup with low comorbidity or who presented to the ED with minor complaints [25, 26] for which we found a comparable group (No Admission cohort) and further classified them as *Stable*,* Increasing*, and *Hyperacute*. Dufour identified a subgroup of frequent users with pulmonary and cardiac conditions [26]; in our study, COPD was a frequent diagnosis in both *Stable* and *Increasing* groups.

Clinical implications

Our study identified a group of patients such as the* Hyperacute *group who could use an alternative care setting to reduce the number of ED visits. Likewise, COPD was a frequent diagnosis and better outpatient management could help to reduce overall ED use.

The reliance on the ED for follow-up care and the use of multiple EDs suggest that the care of this population is fragmented. Return visits for reassessment, imaging, and consultation accounted for 11.3% (5782 visits) of all ED visits in our sample. All trajectories had patients with at least one expected return visit (6.8-20.9%). We did not find literature on the use of the ED for ongoing care and evaluation. Much of the literature has looked at access to primary care as a driver of avoidable ED use [28-30]; however, in our study, expected return visits suggest a lack of access to higher levels of care (e.g., imaging, consultation). In the Admission cohort, 34 % of patients used more than one ED. This cross-facility use constrains the ability of a single institution to identify frequent users and may, in part, be driven by ambulance distribution protocols.

Research implications

Further study to determine whether these trajectories are associated with different levels of adverse outcome may assist ED physicians in identifying patients for admission or earlier community follow-up. We were unable to differentiate trajectory membership based on the variables available to us, and future work could look at chronic disease, disease severity, frailty, and place of residence.

As our dataset contained all insured ED visits in the province, we were able to create a complete portrait of ED use of our study population. Our findings may not be generalizable to other practice settings; however, we demonstrated the utility of GBTM for exploring patterns of use, with potential application in other health systems. While the data were 10 years old, the challenges are likely to be similar and potentially magnified given the increase of ED visits of older adults over time.

The data did not include length of stay for hospital admissions, which would decrease the number of patients available to re-present to the ED in the Admission cohort; however, we believe the shape of trajectories would not change. Transfers could also have impacted the trajectories as these visits would have another visit closely associated in time. The transfer rate was low (4.4% of all visits), and we noted that it had a negligible impact on the model. The dataset was limited to the presenting complaint and discharge diagnosis and did not include past medical history. As a result, the presence of chronic illness may have been underestimated, as conditions not captured in ED discharge diagnoses would not have been identified. A limitation when using administrative data is the potential for misclassification of the diagnostic codes (ICD-10) either from incorrect data entry or from individual institutional coding practices. We elected to retain the original coding rather than aggregating codes in order to avoid introducing bias. There was no information on disease severity or on place of residence (i.e., community dwelling or institution).

## Conclusions

We described distinct trajectories of ED use over time among older adults with three or more visits within a 90-day period. The identified patterns - *Stable*, *Increasing*, and *Hyperacute* - suggest clinically distinct patient subgroups with unique challenges in accessing care, underscoring the potential for trajectory-specific interventions. Although based on data from 2014-2015, the findings remain relevant given the ongoing demographic shift toward an older population and the sustained pressures on ED capacity. By focusing on frequent use over a shorter interval (90 days vs. 365 days), we identified a more clinically relevant subgroup for emergency physicians, offering insights to better identify high-risk patients and to tailor interventions to improve patient care and resource utilization.

## Figures and Tables

**Figure 5:**
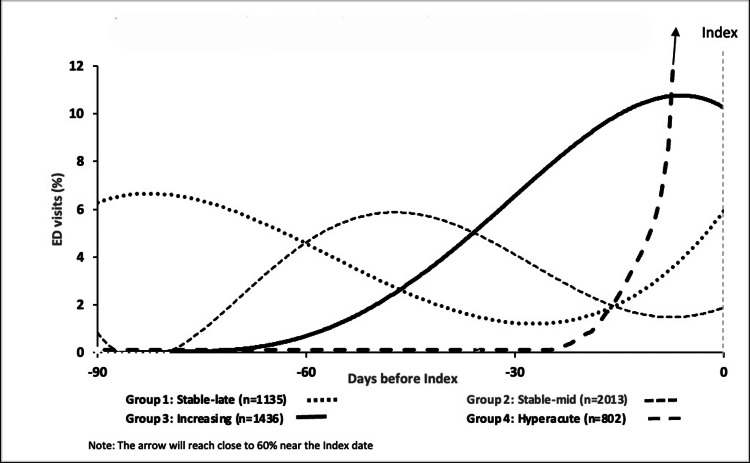
Four-cluster solution: No Admission cohort (n=5,386)

## Appendices

Appendix

We report the details of the data analysis section that were not presented in the manuscript. Also, we present the results of all supplementary analyses.

Data analysis

Model Selection

The model fitting procedure of GBTM is iterative and requires some a priori decisions: the model would have two to five trajectory groups, the estimated percentage of each trajectory must be at least 5%, and the shape of the trajectories would be a polynomial of degree 1 (linear) to 4 (quadratic). We began by assuming a fourth-order polynomial in each group and then simplified the model group-by-group, using the highest value of the Bayesian information criterion (BIC) as the criterion for model selection. However, this was moderated by preference for a useful parsimonious model that fitted the data well. To assess the model adequacy, two measures proposed by Nagin were computed: the average posterior probability of assignment (APPA) and the odds of correct classification (OCC). An APPA of at least 70% and an OCC of at least 5 for each trajectory group suggests a model with good fit [1,2].

The results of the model fitting and adequacy are presented in Tables 4, 5. Figure 5 is the four-cluster solution for the No Admission cohort.

**Table 4:** Trajectory model selection results BIC, Bayesian information criterion For each subsample, the model selected is highlighted

Number of groups	Polynomial order	BIC based on individuals	% estimated in the smallest group	Range of mean posterior probabilities in each group (%)
No Admission (n=5,386)				
2	4,4	-79626	26.5	87-94
3	4,4,4	-78978	17.9	80-88
4	4,4,4,4	-78542	15.3	76-84
5	4,4,4,4,4	-78379	4.4	75-84
2	3,3	-79620	26.1	88-95
3	3,3,3	-78977	19.2	78-86
4	3,3,3,3	-78533	14.8	76-85
5	3,3,3,3,3	-78251	0.5	72-97
Admission (n=5,355)				
2	4,4	-95423	34.1	74-85
3	4,4,4	-95331	11.1	65-73
2	3,3	-95492	33.2	71-86
3	3,3,3	-95369	22.9	65-74
4	-	No solution converge		

**Table 5:** Trajectory model adequacy results P, actual probabilities of patients assigned using the maximum probability rule APP, average posterior probability; OCC, odds of correct classification

Trajectory	Label	Polynomial order	Group membership probabilities (p)	N	P	|p-P| (%)	APP	OCC
No Admission (n=5,386)								
1	Stable	Cubic	0.506	2758	0.512	0.6%	88%	20.8
2	Increasing	Cubic	0.301	1585	0.294	0.7%	78%	20.4
3	Hyperacute	Cubic	0.192	1043	0.194	0.2%	86%	57.4
Admission (n=5,355)								
1	Stable	Quartic	0.659	3618	0.676	1.7%	85%	11.6
2	Increasing	Quartic	0.341	1737	0.324	1.7%	74%	13.9

Sensitivity analysis

Ten samples were generated using a randomly selected index date and the same methodology for model construction was applied to each sample.  To evaluate the robustness of the model, the results from the 10 samples were compared to the whole sample results using three indicators:  the proportion of each group, the visual inspection of shape of each group trajectory, and the model adequacy (APPA and OCC).

For the No Admission cohort, the average proportion of the 10 samples was as follows: group 1, 32.6%; group 2, 18.3%; group 3, 48.8%. The maximum difference between our initial sample and the 10 samples was 1.5%. The shapes were exactly the same, and the performance was consistent for all the 10 samples. Based on this, we concluded our model was robust enough for the non-admitted sample.

For the Admission cohort, the three-group solution showed some important differences. The average proportion of the 10 samples was as follows: group 1, 45.8%; group 2, 22.7%; group 3, 31.7%. The difference between our initial sample and the 10 samples was between 5% and 10%. The shapes of the trajectories were visually different for half of the 10 samples. Finally, the performance of the clustering was borderline for some of the samples. The two-group solution performed better. The average proportion of the 10 samples were as follows: group 1, 36%; group 2, 64%. The difference between our initial sample and the 10 samples was 3.4%. The shapes were similar, and the performance was good for all the 10 samples. (minimum APP=0.72 and minimum OCC=12). The two-group solution was more stable and robust than the three-group solution, and, as a result, the two-group solution was retained.

Supplementary analyses

The results of the supplementary analyses are presented in Tables 6, 7.

**Table 6:** Characteristics of ED visits prior to index visit among Admission and No Admission sub-samples by trajectory group (n=10,741) *Including missing diagnosis

Characteristics	No Admission (n=5,386)						Admission (n=5,355)			
	Stable (n=2,758)		Increasing (n=1,585)		Hyperacute (n=1,043)		Stable (n=3,618)		Increasing (n=1,737)	
Number of visits prior to index visit, n (%)										
3	1717	62%	993	63%	653	63%	2242	62%	1100	63%
4	594	22%	333	21%	217	21%	823	23%	388	22%
5	220	8%	147	9%	74	7%	305	8%	135	8%
6+	227	8%	112	7%	99	9%	248	7%	114	7%
Mean (SD)	3.8 (2.2)		3.8 (1.7)		3.8 (1.6)		3.7 (1.3)		3.7 (1.8)	
Visit without admission (ED), mean (SD)	3.8 (2.2)		3.8 (1.7)		3.8 (1.6)		2.1 (1.4)		2.3 (1.8)	
Visit leading to admission (ADM), mean (SD)	Not applicable						1.6 (0.8)		1.4 (0.6)	
Most frequent sequence of the three closest visits before index visit ("X1"-"X2"-"X3"); X3 is closest to index visit, n (%)										
ED-ED-ADM	Not applicable						865	22%	310	18%
ED-ADM-ED							719	20%	295	17%
ADM-ED-ED							420	12%	510	29%
ED-ADM-ADM							501	14%	65	4%
ADM-ED-ADM							306	8%	165	10%
ED-ED-ED							166	5%	228	13%
ADM-ADM-ADM							357	10%	19	1%
ADM-ADM-ED							284	8%	145	8%
Time between the closest visit to the index visit (days), mean (SD)	24.6 (21.2)		9.8 (8.0)		1.9 (1.3)		24.7 (18.3)		9.3 (7.6)	
Time between the closest visit (with admission) to the index visit (days), mean (SD)	Not applicable						40.7 (22.7)		31.7 (23.3)	
Time between the closest visit (without admission) to the index visit (days), mean (SD)	24.6 (21.2)		9.8 (8.0)		1.9 (1.3)		36.7 (25.4)		11.6 (10.0)	
Same discharge diagnosis* at index visit as previous visit, n (%)	450	16%	352	22%	425	41%	544	15%	335	19%
Same facility at index visit as previous visit facility, n (%)	2311	84%	1382	87%	949	91%	3076	85%	1434	83%
Number of different hospitals before index hospital visit, n (%)										
0	1964	71%	1173	74%	862	83%	2426	67%	1098	63%
1	643	23%	337	21%	149	14%	987	27%	545	32%
2	127	5%	66	4%	32	3%	185	5%	87	5%
3+	24	1%	9	1%	0	0%	20	1%	5	0%

**Table 7:** Most important diagnoses* for trajectory groups in the No Admission and Aadmission sub-sample (diagnoses are sorted alphabetically according to the ICD-10) *Most important diagnoses were chosen from PLS regression. Relevant odds ratios (OR) were defined as OR>1.5 or OR<0.67 from multivariable model regression; significant OR (at 0.05) are in bold font. COPD, chronic obstructive pulmonary disease; PLS, partial least squares

Variables	Stable	Increasing	Hyperacute	Increasing than Stable	Hyperacute than Stable	Hyperacute than Increasing
	%	%	%	OR [95%]	OR [95%]	OR [95%]
No Admission (n=5,386)	(n=2,758)	(n=1,585)	(n=1,043)	Nominal logistic regression		
Diagnoses more frequent in increasing/hyperacute trajectories				Multivariable model		
Cellulitis (L039)	8.9%	13.8%	36.1%	-	3.81 [3.14; 4.62]	2.67 [2.18; 3.26]
Bursitis, unspecified (M7199)	0.4%	1.1%	1.9%	2.18 [1.01; 4.69]	2.60 [1.19; 5.67]	-
Open wound lower limb (T131)	0.8%	1.7%	0.7%	1.65 [0.93; 2.91]	-	-
Follow-up exam unspecified treatment (Z099)	2.6%	4.4%	4.5%	1.54 [1.10; 2.16]	-	-
Other specified medical care (Z5188)	8.3%	7.1%	12.6%	-	-	1.62 [1.23; 2.14]
Diagnoses less frequent in increasing/hyperacute trajectories						
Anemia (D649)	2.5%	1.4%	0.3%	0.52 [0.32; 0.85]	0.11 [0.03; 0.35]	0.21 [0.06; 0.71]
Fluid overload (E877)	1.7%	0.9%	0.2%	0.52 [0.28; 0.95]	0.13 [0.03; 0.55]	0.25 [0.06; 1.12]
Atrial fibrillation (I480)	2.6%	2.3%	0.7%	-	0.30 [0.13; 0.66]	0.33 [0.15; 0.76]
Bronchitis, acute (J209)	4.2%	3.0%	1.4%	-	0.38 [0.22; 0.67]	0.51 [0.28; 0.92]
COPD with respiratory infection (J440)	3.2%	1.5%	0.6%	0.45 [0.28; 0.72]	0.26 [0.11; 0.61]	0.57 [0.22; 1.45]
COPD exacerbation (J441)	4.4%	4.0%	1.3%	-	0.47 [0.26; 0.85]	0.43 [0.23; 0.80]
Constipation (K590)	4.6%	5.1%	2.2%	-	0.64 [0.40; 1.02]	0.54 [0.34; 0.88]
Back pain (M545)	7.2%	6.3%	3.1%	-	0.47 [0.32; 0.70]	0.55 [0.36; 0.83]
Myalgia (M7919)	1.9%	2.4%	0.4%	-	0.21 [0.07; 0.59]	0.17 [0.06; 0.48]
UTI - Urinary tract infection (N390)	10.5%	9.3%	4.9%	-	0.49 [0.36; 0.67]	0.56 [0.40; 0.78]
Palpitations (R002)	2.3%	1.3%	0.5%	0.63 [0.38; 1.04]	0.29 [0.11; 0.74]	0.46 [0.17; 1.24]
Cough (R05)	3.0%	1.8%	1.0%	0.66 [0.43; 1.02]	0.44 [0.22; 0.87]	-
SOB - Dyspnea (R060)	7.5%	6.9%	1.8%	-	0.37 [0.23; 0.61]	0.36 [0.22; 0.59]
CP - Chest pain (R074)	12.3%	9.3%	4.4%	-	0.43 [0.31; 0.59]	0.56 [0.40; 0.79]
Abdominal pain / Colic (R104)	14.1%	11.2%	6.3%	-	0.54 [0.41; 0.72]	-
Weakness / Fatigue (R53)	8.8%	7.2%	4.2%	-	0.62 [0.44; 0.87]	-
Syncope / Vasovagal (R55)	3.7%	2.3%	1.1%	0.64 [0.43; 0.94]	0.34 [0.18; 0.65]	0.54 [0.27; 1.08]
Open wound lower limb (T131)	0.8%	1.7%	0.7%	-	0.38 [0.16; 0.93]	0.23 [0.10; 0.55]
Admission (n=5,355)	(n=3,618)	(n=1,737)	-	Logistic regression		
Diagnoses for ED visits resulting in an admission				Multivariable model		
Diagnoses more frequent in increasing trajectories						
Hypo-osmolality and hyponatremia (E871)	0.7%	1.2%	-	1.65 [0.92; 2.97]	-	-
Influenza (J118)	0.8%	1.3%	-	1.56 [0.89; 2.74]	-	-
Diverticular disease of intestine (K578)	0.5%	1.0%	-	2.03 [1.03; 3.98]	-	-
Renal colic (N23)	0.3%	0.8%	-	2.16 [0.96; 4.83]	-	-
Diagnoses less frequent in increasing trajectories						
Clostridium difficile (A047)	2.1%	1.2%	-	0.53 [0.32; 0.86]	-	-
Malignant neoplasm of colon (C189)	0.8%	0.2%	-	0.20 [0.06; 0.65]	-	-
Malignant neoplasm of prostate (C61)	0.4%	0.2%	-	0.33 [0.10; 1.15]	-	-
Anemia (D649)	2.7%	1.8%	-	0.64 [0.43; 0.97]	-	-
Hypotension (I959)	1.0%	0.6%	-	0.60 [0.30; 1.17]	-	-
COPD with respiratory infection (J440)	4.4%	2.5%	-	0.65 [0.46; 0.92]	-	-
COPD exacerbation (J441)	7.1%	4.1%	-	0.59 [0.45; 0.78]	-	-
Cirrhosis of liver (K746)	0.9%	0.5%	-	0.53 [0.25; 1.13]	-	-
Cholangitis or cholecystitis without obstruction (K8050)	0.5%	0.2%	-	0.30 [0.09; 1.04]	-	-
Urinary tract infection (N390)	3.0%	1.9%	-	0.59 [0.39; 0.87]	-	-
Somnolence (R400)	0.5%	0.1%	-	0.23 [0.05; 0.99]	-	-
Dizziness and giddiness (R42)	0.5%	0.2%	-	0.30 [0.09; 1.01]	-	-
Fever (R509)	2.7%	1.9%	-	0.65 [0.44; 0.97]	-	-
Convulsions (R5688)	0.5%	0.2%	-	0.39 [0.13; 1.15]	-	-
Multiple superficial injuries (T009)	0.7%	0.4%	-	0.51 [0.21; 1.27]	-	-
Psychosocial circumstances (Z659)	0.5%	0.2%	-	0.41 [0.14; 1.20]	-	-
Diagnoses for ED visits without admission				Multivariable model		
Diagnoses more frequent in increasing trajectories						
Mental and behavioural disorder (F100)	0.3%	0.6%	-	1.77 [0.75; 4.17]	-	-
Major Depressive Disorder (F329)	0.5%	0.8%	-	1.66 [0.81; 3.42]	-	-
Supraventricular tachycardia (I471)	0.2%	0.5%	-	2.67 [0.99; 7.21]	-	-
Ulcerative colitis (K519)	0.5%	0.9%	-	1.65 [0.83; 3.26]	-	-
Cellulitis (L039)	3.4%	6.0%	-	1.80 [1.38; 2.36]	-	-
Urinary calculus (N209)	0.3%	0.6%	-	1.76 [0.72; 4.32]	-	-
Renal Colic (N23)	0.4%	0.8%	-	1.58 [0.73; 3.44]	-	-
Hemoptysis (R042)	0.3%	0.5%	-	2.21 [0.87; 5.62]	-	-
Dizziness and Giddiness (R42)	0.6%	1.0%	-	1.67 [0.89; 3.11]	-	-
Abnormal finding of blood chemistry (R799)	0.3%	0.6%	-	1.96 [0.83; 4.65]	-	-
Psychosocial circumstances (Z659)	0.3%	0.7%	-	2.02 [0.89; 4.53]	-	-
Diagnoses less frequent in increasing trajectories						
Acute upper respiratory infection (J069)	1.5%	0.7%	-	0.46 [0.25; 0.87]	-	-
COPD with respiratory infection (J440)	2.4%	1.4%	-	0.65 [0.41; 1.02]	-	-
Disorientation (R410)	0.7%	0.4%	-	0.54 [0.22; 1.31]	-	-
Unspecified injury of head (S009)	0.5%	0.2%	-	0.32 [0.09; 1.08]	-	-
Concussion (S060)	1.1%	0.5%	-	0.45 [0.22; 0.94]	-	-
Other injuries of upper limb (T110)	0.4%	0.2%	-	0.39 [0.11; 1.33]	-	-
